# Severity of Unconstrained Simultaneous Bilateral Slips: The Impact of Frontal Plane Feet Velocities Relative to the Center of Mass to Classify Slip-Related Falls and Recoveries

**DOI:** 10.3389/fpubh.2022.898161

**Published:** 2022-07-11

**Authors:** Abderrahman Ouattas, Corbin M. Rasmussen, Nathaniel H. Hunt

**Affiliations:** Department of Biomechanics, Division of Biomechanics, University of Nebraska Omaha, Omaha, NE, United States

**Keywords:** biomechanics, mediolateral, diverse-slips, recovery, response, gait, falls, fall-prevention

## Abstract

Targeted interventions to prevent slip-related falls may be informed by specific kinematic factors measured during the reactive response that accurately discriminate recoveries from falls. But reactive responses to diverse slipping conditions during unconstrained simultaneous bilateral slips, which are closely related to real-world slips, are currently unknown. It is challenging to identify these critical kinematic factors due to the wide variety of upper and lower body postural deviations that occur following the slip, which affect stability in both the sagittal and frontal planes. To explore the utility of kinematic measurements from each vertical plane to discriminate slip-related falls from recoveries, we compared the accuracy of four Linear Discriminant Analysis models informed by predetermined sagittal or frontal plane measurements from the lower body (feet velocities relative to the center of mass) or upper body (angular momentum of trunk and arms) during reactive responses after slip initiation. Unconstrained bilateral slips during over-ground walking were repeatedly administered using a wearable device to 10 younger (24.7 ± 3.2 years) and 10 older (72.4 ± 3.9 years) adults while whole-body kinematics were measured using motion capture. Falls (*n* = 20) and recoveries (*n* = 40) were classified by thresholding the dynamic tension forces measured in an overhead harness support system and verified through video observation. Frontal plane measurements of the peak feet velocities relative to the center of mass provided the best classification (classification accuracy = 73.3%), followed by sagittal plane measurements (classification accuracy = 68.3%). Measurements from the lower body resulted in higher accuracy models than those from the upper body, but the accuracy of all models was generally low compared to the null accuracy of 66.7% (i.e., predicting all trials as recoveries). Future work should investigate novel models that include potential interactions between kinematic factors. The performance of lower limb kinematics in the frontal plane in classifying slip-related falls demonstrates the importance of administering unconstrained slips and measuring kinematics outside the sagittal plane.

## Introduction

Fall rates increase significantly after the age of 65 (1). For older adults, falls are the greatest cause of injury and injury-related mortality (2). Fall-related injury rates are higher in older fallers compared to younger adults (2), with at least 25% suffering an injury (3). Slips were reported to be the second leading cause of falls, accounting for ~25% of all fall instances and as much as 50% of all injuries from falls (4). Based on the most recent published report by the US Bureau of Labor Statistics, falls, trips, and slips were responsible for the second highest proportion of nonfatal occupational injuries and illnesses involving days away from work in 2020 (211,640 of 1,176,340 cases) (5). Slips alone accounted for over 39% of overall recorded falls on the same level (50,100 of 127,680 falls) (5). The annual fall-related injury and mortality rates are growing year over year (6), increasing the importance of identifying effective strategies to resist slips and prevent falls. However, discovering good slip resistance strategies is a challenge because the mechanics of slips vary widely with respect to the slipping direction of each foot and the loss of upright trunk posture across the frontal and sagittal planes (7, 8). A reactive balance control strategy that is effective at resisting one slip may be ineffective at resisting another (8). As a result, identifying specific biomechanical factors that are capable of discriminating slip-related falls from recoveries is challenging given the kinematic heterogeneity of slips.

The diversity of observed slip mechanics likely originates from the complex interaction between the walking surface, the applied forces through the foot that change direction across stance phase, and the unanticipated time of traction loss. The natural diversity of slips is revealed in the laboratory by administering repeated simulated slips that are unconstrained, where the slipping foot is not restricted to slide only in the anteroposterior direction. These unconstrained slips often show a strong lateral component in the displacement of the slipping foot (7–9). Resisting an unconstrained slip may require complex motor skills that are sensitive to the environmental context (e.g., surface slope and available friction) and specific mechanics of the slipping episode. In general, to accomplish a successful recovery, one needs to restore body weight support before collision with the ground and arrest the rotation of the trunk through angular impulses from ground reaction forces, potentially assisted by the transfer of angular momentum from the trunk to the arms to maintain an upright trunk posture (10). However, a singular kinematic strategy for resisting slips is unlikely to be effective for all cases where the feet can slip anteriorly and/or laterally and loss of balance as a result can occur backwards or to the side.

**Figure 1:**
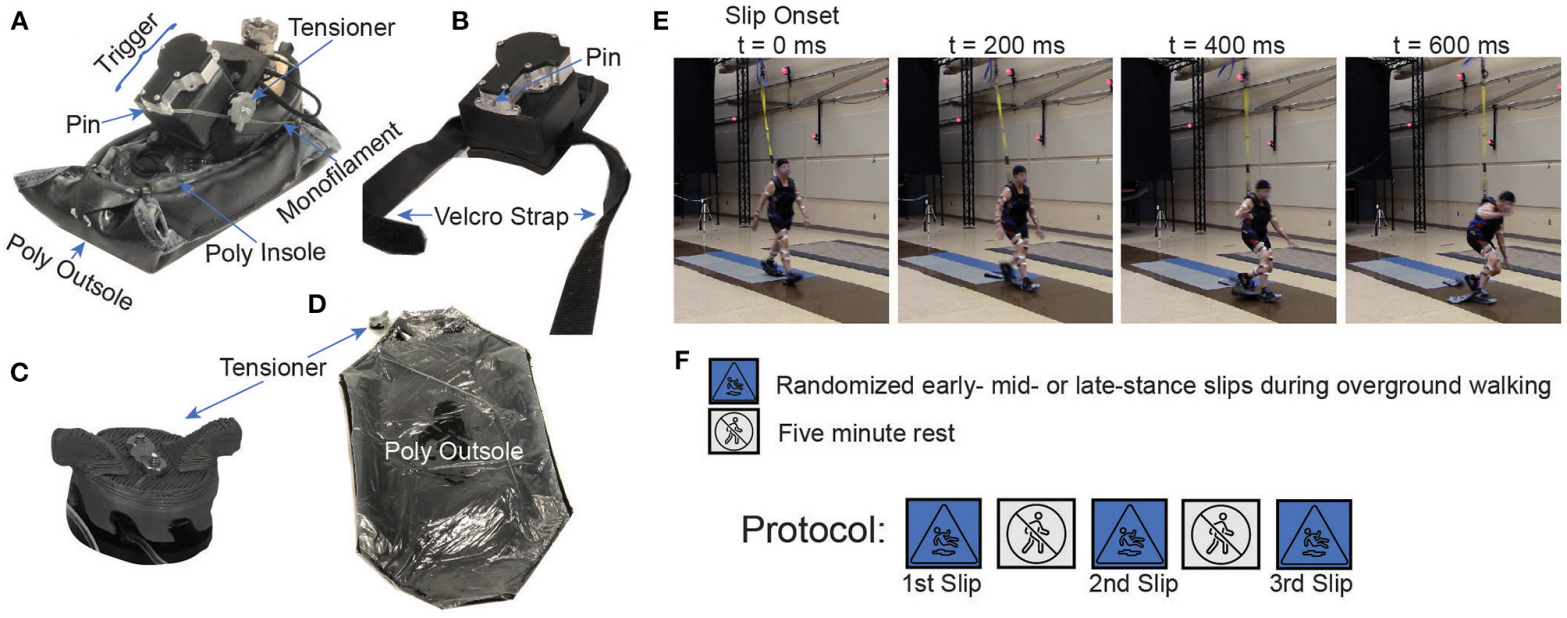
**(A)** Wearable Apparatus for Slipping Perturbations (WASP) assembly fitted over an athletic shoe worn over a prosthetic foot. **(B)** The WASP trigger device that was custom built in-lab using metal additive manufacturing; the WASP uses a pin and follower mechanism, and when it triggers, the pin draws backward and releases the monofilament holding the outsole, hence detaching the external (outsole) from the internal (insole) soles, causing a rapid decrease in friction. **(C)** The tensioner adjusts the monofilament tension holding the outsole based on the participant's shoe size. **(D)** Outsole consists of a layer of rubber, and a layer of PTFE, (Teflon), coated with WD-40 oil to ensure reduced friction with the insole. **(E)** Photo sequence shows a representative trial of a bilateral slips administered to the dominant (right foot in this case) limb during early stance, which automatically induces a late stance slip on the non-dominant foot (left). The photos are taken at 200, 400, and 600 ms post slip onset. **(F)** Protocol Outline. Participants completed three randomized unconstrained simultaneous bilateral slips trials of early stance (ES), mid stance (MS), and late stance (LS). Participants rested while seated for 5 min between trials.

The diversity of slip mechanics observed during unconstrained slips in the lab is consistent with the limited data available on the biomechanics of slip-related falls in the community. For example, 49% of observed falls due to outdoor slips occurred over diverse surface conditions (11), and a third of all observed falls in a sample of community dwelling older adults had a strong lateral component (12). However, some common methods for simulating slips in the lab do not generate unconstrained slips. Treadmill-belt perturbations (13–16) or sliding platforms embedded in the floor (9, 15, 17–19) during over-ground locomotion restrict motion of the slipping foot to the anteroposterior direction and therefore may not reproduce the diversity of slip mechanics across both sagittal and frontal planes. Other slip simulation methods employing low friction sheets or lubricants applied unexpectedly in a person's walking path (9, 10, 20–22) do produce unconstrained slips, however the location of the slip is predictable after the first occurrence, making the delivery of repeated unpredictable slips challenging. To simulate unconstrained slips that occur at unpredictable times and locations in the lab, our research group developed the Wearable Apparatus for Slipping Perturbations (WASP, Figure 1). This device functions like a remote-controlled banana peel (7) that can simulate slips on a wide range of walking surfaces (e.g. treadmills, slopes) and tasks (e.g. turning). Here, we employ the WASP to repeatedly administer slips to both the dominant and non-dominant lower limbs simultaneously during early, mid-, and late phases of stance while walking a straight path.

Due to the diversity of slips, the biomechanical factors that are causally related to the outcome (i.e. falls or recoveries) are unknown. Here, we compared the accuracies of four biomechanical models to discriminate between falls and recoveries during simultaneous unconstrained bilateral slips. The biomechanical measurements informing the four models are separated along two dimensions: (1) upper body versus lower body, and (2) sagittal plane versus frontal plane. The first and second models feature lower body biomechanics, specifically sagittal and frontal plane peak dominant and non-dominant feet velocities relative to the Center of Mass (CoM) during the slip. The third and fourth models feature upper body biomechanics, specifically sagittal and frontal plane peak trunk and arm (dominant and non-dominant) angular momenta during the slip. Our four biomechanical models were inspired by evidence from literature despite them using unilateral slips. Troy et al. (21) showed an increased probability of recovery when lateral foot velocity and displacement are closer to that of the CoM. Wang et al. (17) also showed that foot placement relative to the CoM affects the probability of a fall . Therefore, we incorporated lower body biomechanics in the form of feet velocities relative to the CoM into our models since keeping the base of support under the CoM is likely vital to avoid a fall, especially when individuals transition from a walking to a sliding technique. In terms of upper body biomechanics, Grabiner et al. (23) showed that trunk angular velocities in the frontal plane were higher in fall trials experienced by older adults . In addition, Troy et al. (10) showed that rapid shoulder flexion reduces trunk extension to help with the recovery, a phenomena widely seen after slip initiation when the trunk is rapidly extended. Consequently, we incorporated trunk and arm angular momenta into our upper body models since minimizing trunk deviations from vertical can help prevent a fall (24).

As a first step, it is vital to understand how individuals behave when they are exposed to unconstrained simultaneous bilateral slips and which biomechanical factors within each plane of motion contribute more to falls and recoveries. The objective of this study was to examine the importance of frontal plane biomechanics in both testing (e.g., in-lab slips) and analyses (e.g., biomechanical models) when individuals are exposed to unconstrained bilateral slips. We aimed to determine if frontal plane biomechanical factors can better discriminate between falls and recoveries compared to sagittal plane factors and if lower body biomechanical factors can better classify falls and recoveries compared to upper body factors. We first hypothesized that, due to the active control required to stabilize lateral balance during walking (25) due to sagittal slips, frontal plane biomechanical variables would be better predictors of falls and recoveries compared to sagittal plane biomechanical variables. This hypothesis is further motivated by the high prevalence of real-world falls that occur laterally due to sagittal slips and trips (12), and our previous findings that show the foot sliding in the frontal and sagittal planes due to unconstrained in-lab slips (8). Second, we hypothesized that slipping feet velocities relative to the CoM would be better predictors of falls and recoveries compared to upper body angular momentum regardless of the plane of motion. This hypothesis is motivated by the fundamental role of applied forces through the feet to produce the reaction forces and impulses required for body weight support and trunk posture maintenance.

## Methods

### Participants

Ten younger and 10 older adults participated in the current research study (Table 1). Participants were excluded if they self-reported (1) uncontrolled hypertension, (2) peripheral arterial disease, (3) vertigo, (4) Meniere's disease, (5) chronic dizziness, (6) history of a recent back or lower extremity injury, (7) surgery that affects the participant's mobility, and (8) any neurological disease or impairment that limits their ability to walk (stroke, Parkinson's disease, multiple sclerosis). Younger participants were recruited through the university's newsletter sent to students and staff, ClinicalTrials.gov, word of mouth, and flyers posted across the Omaha community. Older participants were recruited from the university, community wellness centers, and from the community dwelling older adult population *via* flyers and word of mouth.

**Table 1:** Participant demographics.

**Participants**	**Age (years)**	**Height (m)**	**Weight (kg)**	**BMI (kg/m^**2**^)**	**Gender**
Younger adults (*n* = 10)	24.70 (3.16)	1.71 (0.09)	73.68 (12.93)	25.08 (3.70)	5F/5M
Older adults (*n* = 10)	72.40 (3.86)	1.68 (0.12)	79.74 (14.86)	27.89 (2.69)	5F/5M

*Mean (standard deviation)*.

The study and procedures involved, including the risks, were explained and discussed with all participants prior to their involvement in the research study. All procedures were explained in detail to each participant over the phone prior to their visit and were reviewed during the initial visit to the Biomechanics Research Building at the University of Nebraska at Omaha before informed consent was obtained. The study was approved by the University of Nebraska Medical Center's Institutional Review Board (IRB# 877-18-EP).

### Experimental Protocol

To investigate slips under diverse slipping conditions, we used our custom WASP device (Figure 1A). The design and function of this device has been reported previously (7). Briefly, the WASP is designed to be worn over standard athletic shoes and triggered wirelessly by the experimenter. The entire device weighs 631 g, with 210 g contributed by the trigger mechanism (Figure 1B), 161 g by the polyethylene sock worn under the participant's shoe (Poly Insole, Figure 1A), and 260 g by the detachable outsole (Figure 1D). The trigger mechanism that sits atop the wearer's foot (Figure 1B) is 9 cm long, 6.75 cm wide, and 6 cm tall. In a previous validation study, we compared lower limb gait kinematics between walking with and without the WASP device attached to one's feet. Through this comparison, we found that gait kinematics were minimally affected by the device, with the greatest change of 3.4° root-mean-square error seen at the ankle (7). Before the WASP is triggered, the participant walks with a high friction interface between the rubber outsole (Poly Outsole, Figure 1A) and laboratory floor. When the WASP is triggered wirelessly *via* Bluetooth connection (Figure 1B), it retracts a pin with a cam-and-follower mechanism, which separates the polyethylene (Poly) sock worn over the participant's shoe (Poly Insole, Figure 1A) from the lubricated Poly outsole (Figure 1D). This suddenly reduces the available friction underfoot (7) (Supplementary Video 1), allowing the sock to slide over the lubricated outsole and onto the lab floor (Supplementary Video 1). Participants wore the WASP during all walking trials. To quantify the potential difference in available friction between sliding over the outsole versus over the lab floor, we measured the Dynamic Coefficient of Friction (DCoF) of the interface of the sock with each surface. We measured the acceleration of an unworn WASP (α_WASP_) as it slid along a sloped surface of angle θ on either a floor tile surface or a Poly surface (Figure 1E). Each experiment consisted of 10 trials, and only the insole was coated with WD-40^®^ (low friction lubricant) between each trial. Moreover, we measured the WASP's acceleration as it slid on the Poly surface with no added WD-40 between trials to measure the influence of lubricant amount on DCoF (i.e. WD-40 was only added before the first trial). The DCoF was then calculated based on the shoe and gravitational (g) accelerations measured from release until impact with the floor (Equation 1).


(1) $$\begin{array}{r} DCoF=\;\frac{g\sin\theta -a_{WASP}}{g\cos\theta } \end{array}$$

DCoF reduced from a coefficient of approximately μ = 0.5 (min coefficient of friction of slip resistant walking surfaces in the courts of law in the US) (26) to a coefficient close to that experienced between shoes and ice (μ = 0.05) (27) (*p* < 0.001) and lower than the minimum available DCoF during walking (μ = 0.2) (28) (Poly to Poly μ = 0.14 ± 0.04, Poly to Floor μ = 0.12 ± 0.01). The DCoF was slightly reduced to μ = 0.13 ± 0.02 when measured between two Poly interfaces without adding WD-40, however the difference was not statistically significant (*p* = 0.53), indicating that the amount of lubricant has little influence on DCoF.

The research experiment took place in the main Motion Analysis Laboratory of the Biomechanics Research Building (Figure 1E). Each participant performed three unconstrained simultaneous bilateral slips administered randomly at either early (0–33.3% of stance), mid- (33.4%−66.7% of stance), or late (66.8%−100% of stance) stance phase of the dominant foot (Figure 1F). After obtaining informed consent, demographic information (i.e. age, gender) and anthropometric measurements were collected. Participants were asked to fill out a health history questionnaire and to report their history of falls before the intervention. Each participant was fitted with a one-piece compression uniform to ensure reliable reflective marker readings, standardized athletic shoes to normalize the effects of shoe type on slip recovery, a safety harness connected to an overhead rail system, and a WASP device on each foot. During the slip perturbation trials, participants walked back and forth across a 10-meter pathway within a large motion capture area (Figure 1E). Prior to performing any trials, participants were allowed to “sit” in the harness to experience how it feels when it catches them and to make sure their knees would not contact the floor. Literature have shown that walking speed is highly correlated with variations in whole body CoM trajectories, joint moments, joint angles, intersegmental coordination, and overall gait behavior during walking on a slippery surface (29). Therefore, all participants walked at a constant gait velocity of 1.3 ± 0.1 m^.^s^−1^ before the slip, a typical comfortable speed for younger and older adults (30). Walking speed was monitored using a Dashr timing system (Dashr, Lincoln, NE, USA). Participants were informed that a slip may occur between 1 and 3 min of walking and that if slipped, they should attempt to do their best to not fall (e.g. load all their body weight on the safety harness) and stop walking. Participants were then slipped without warning at a randomly chosen time and location between 1 and 3 min after the initiation of walking. Slips were administered to both feet simultaneously at either early, mid, or late stance phase. Because the prescribed slip onset phases apply to the dominant foot only and friction was reduced under both feet simultaneously, the reader should note that the non-dominant foot is perturbed during a different stance phase than the dominant foot. For example, an early stance slip of the dominant foot will simultaneously trigger a late stance slip of the non-dominant foot, and vice versa. After each slip, participants rested in a seated position for 5 min. During this rest period, the WASP devices were reattached on the participant's shoes for the next trial.

### Biomechanical Measurements

A whole-body set of 83 retroreflective markers were placed on the legs, arms, torso, and head to obtain whole-body kinematic data (Supplementary Figure). The individual markers were fixed to anatomical landmarks to define the position of each segment of the participants' bodies. The clusters were attached to the participants' thighs, shanks, upper arms, and forearms.

Kinematics were recorded using a 17-high speed infrared camera motion capture system (Motion Analysis Corp.; Santa Rosa, CA, USA) to collect 3-D marker positions sampled at 100 Hz. All kinematic data were interpolated with a maximum gap of 10 frames and a polynomial order of 3, and low-pass filtered at 6 Hz using a fourth-order Butterworth filter. Kinetics were collected using a load cell attached to the harness, with a maximum weight capacity of 500 kg (HT Sensor Technology Co. LTD; Xi'an, China). Kinetic data were sampled at 80 Hz, and low-pass filtered at 8 Hz using a fourth-order Butterworth filter. Kinematic and kinetic data were filtered in accordance with previous work (31–34). Data processing was completed using Cortex 6 software (Motion Analysis Corp.; Santa Rosa, CA, USA). Visual3D software (C-Motion Inc.; Germantown, MD, USA) was used to analyze kinematics and kinetics.

All biomechanical data (Table 2) were analyzed from slip onset until both slipping feet reached a complete stop. This timeline is referred to as “the slipping timeline”. Load cell data quantifying harness assistance were used as the gold standard measure to represent slip severity (Table 2). A trial was classified as a fall if the load cell in the harness system measured a peak force in excess of 30% of the participant's body weight (34). Any load lower than 30% of body weight was considered a recovery. Three slip trials were missing load cell data, however upon video observation we concluded that they were obviously not falls and therefore classified them as recoveries. The whole-body CoM position was derived from the weighted mean position of all individual segment CoM in our 15-segment model (two feet, two shanks, two thighs, one pelvis, one trunk, one head, two upper arms, two forearms, two hands). Peak dominant and non-dominant feet velocities relative to the CoM (*F*_*t*_*v*_*CoM*_) were extracted in both frontal (ML) and sagittal (AP) planes during the slipping timeline. The global maxima and global minima were analyzed; it is worth noting here that both maxima and minima can have either negative or positive values. Peak upper body angular momenta (L) were also extracted in both frontal and sagittal planes during the slipping timeline. In this calculation of whole-body angular momentum about the body's center of mass, (L), the body center of mass is considered the origin (Table 2). The bold symbol *r* represents the displacement vector from the body center of mass to the segment center of mass. The bold symbol *v* represents the velocity vector of the segment center of mass with respect to the body center of mass. The bold symbol *m*_*n*_ is the mass of the *n*th segment, and *I*_*n*_ and ω_*n*_ are the moment of inertia and angular velocity of *n*th segment about its own CoM, respectively. Angular momentum has been normalized to each participant's mass and height. Segment angular velocities (ω_*n*_) were calculated as the derivative of the segment's angle. All segment parameter values, including *m*_*n*_, were estimated from the whole body mass of each individual using predefined methods reported by Dempster (35). The moments of inertia *I*_*n*_ and CoM for each segment were calculated using validated experimental methods by Hanavan (36). Angular momentum directions are defined in Table 2 [Y = Anteroposterior (frontal plane), X = Mediolateral (sagittal plane), Z = vertical (transverse plane)]. The global maxima and minima were extracted in both frontal and sagittal planes for each of three upper body segments: (1) trunk, (2) dominant arm (including forearm, upper arm, and hand segments), and (3) non-dominant arm.

**Table 2:** Analyzed biomechanical data.

**Kinetics kinematics**	**Name**	**Description**	**Sign**	**Calculation**	**Units**
Kinetics	Harness Assistance	Load cell data	⊕= 0–100% of body weight	Measured	% Body weight
Kinematics	Peak dominant & non-dominant feet velocities relative	Maxima (peak) AP *Ft*_*v*_*CoM*__ (⊕ or ⊖)	⊕= Feet are faster and moving away from the CoM	*Ft*_*v*_*CoM*__ = *Ft*_*v*_−*CoM*_*v*_	m·s^−1^
	to CoM *Ftv*_*CoM*_	Minima (Min) AP *Ft*_*v*_*CoM*__ (⊕ or ⊖)	⊖= Feet are slower, and the CoM is moving toward the foot		
		Maxima (Peak) ML *Ft*_*v*_*CoM*__(⊕ or ⊖)	⊕= Feet are moving away laterally from CoM		
		Minima (Min) ML *Ft*_*v*_*CoM*__ (⊕ or ⊖)	⊖= Feet are moving inward medially toward CoM		
	Peak Upper Body Angular Momentum *L*	Maxima (Peak) sagittal plane L ⊕	⊕= Backward Momentum	$\text{L}=\overset{N}{\underset{n=1}{\sum }}m_{n}\left({\text{r}}_{\text{Co}{\text{M}}_{\text{n}}}\times {\text{v}}_{\text{Co}{\text{M}}_{\text{n}}}\right)+I_{n}\cdot \omega_{n}$	kg·m^2^·s−1
		Minima (Min) sagittal plane L ⊖	⊖= Forward Momentum		
		Maxima (Peak) frontal plane L ⊕	⊕= Counter-clockwise rotation		
		Minima (Min) frontal plane L ⊖	⊖= Clockwise rotation		

### Statistical Analyses

Descriptive statistics were calculated for all variables and groups. Q–Q plots and Shapiro-Wilk tests were used to analyze all data for normality. Fisher's Linear Discriminant Analysis (LDA) was used to detect if frontal plane biomechanical variables are better discriminators of falls and recoveries compared to sagittal plane variables and if feet velocities relative to the CoM can better classify falls and recoveries compared to upper body angular momentum. LDA assumes that: (1) the predictors are independent, (2) group memberships are mutually exclusive (i.e. participants cannot be fallers and non-fallers at the same time, at least for each slip onset phase), (3) there are no outliers as this model is very sensitive to outliers, (4) the predictors are normally distributed, (5) within group variance matrices are equal across groups, (6) there is no multicollinearity; predictor variables cannot be highly correlated with one another, and (7) in terms of sample size, the smallest group must exceed the number of predictor variables (ideally, there should be five times the number of observations than predictors). To meet the previous assumptions, a backward stepwise elimination method was used to enter predictors that reduce Wilks' Lambda with probability of *F* ≤ 0.05 and remove predictors with probability of *F* ≥ 0.1 before running the LDA model. Predictors that reduce Wilks' Lambda with probability between 0.05 and 0.1 were only entered in the first step and removed in later steps. The final step will only enter predictors that reduces Wilks' Lambda with probability of *F* ≤ 0.05. Wilks' Lambda outputs the total unexplained variance within the classifiers, and it is used to measure how effectively LDA classifies each group using the entered predictors – smaller values indicate the LDA function is superior in discriminating between classifiers. LDA uses dimensionality reduction analysis to distinguish between two or more classes based on Gaussian and homoscedastic variables, through correct classification of participants values' receiver operating characteristic curve. LDA classifies falls and recoveries based on the receiver operating characteristic's sensitivity and specificity; sensitivity is the portion of recovery trials correctly classified (true positive), and specificity is the portion of the fall trials correctly classified (true negative).

Effect sizes were calculated based on the optimal data analysis paradigm using a measure called the effect size for sensitivity, which is based on LDA's classification accuracy (37). The effect size for sensitivity is calculated based on Equation 2, which requires the mean classification accuracy of both classes (falls and recoveries). Effect size for sensitivity is restricted between 100, where all trials are correctly classified, and −100, where all trials are classified incorrectly, with a value of 0 indicating that trials were classified due to chance. The strength of effect size for sensitivity can be interpreted as follows: values between 0.1 and 24.9 represent a weak effect, between 25 and 49.9 a moderate effect, between 50 and 74.9 a strong effect, and between 75 and 100 a very strong effect.


(2) $$\begin{array}{r} Effect\;Size\;for\;Sensitivity\left(ESS\right)=10\cdot \frac{Mean\;Sensitivity-50}{50} \end{array}$$

The reason we chose only two outcomes (fall or recovery) for LDA is because we had three missing values, and such a limitation breaks one of the assumptions of LDA. Custom MATLAB codes (MathWorks, R2021a, Natick, MA, USA) and SPSS (IBM, v.26, Armonk, NY, USA) were used for statistical analyses and data visualization, and the significance level was set at *p* = 0.05.

## Results

**Figure 2:**
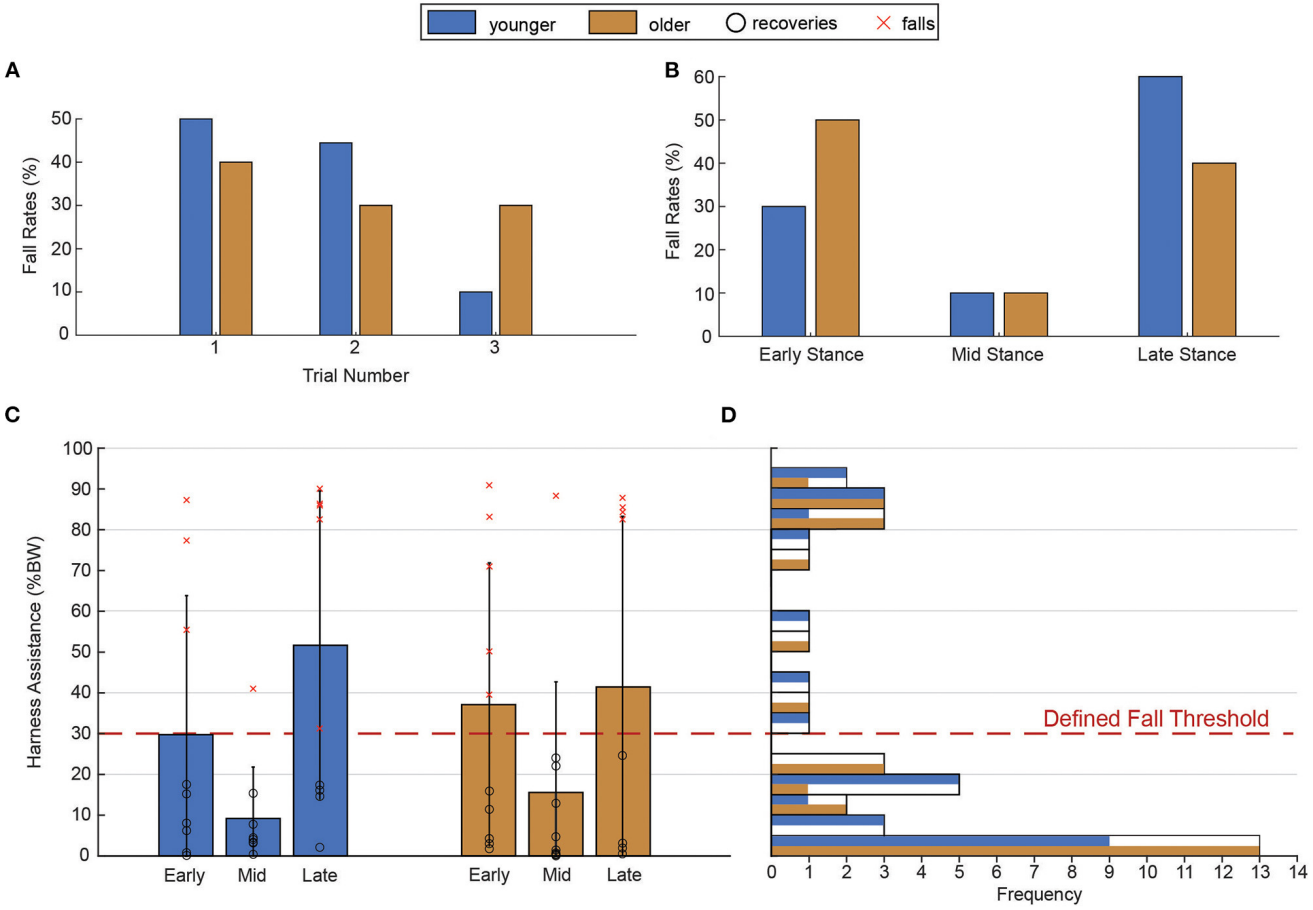
Fall rates disaggregated by age group, trial, stance phase of slip onset, and level of harness assistance. **(A)** Fall Rate (#Falls/Overall Trials) is presented for each trial and between age group. **(B)** Fall Rate presented for each slip onset phase and between age groups. **(C)** Here we demonstrate the change in harness assistance, presented in the x-axis, between younger and older adults across the three different slip onset phases: Early Stance, Mid Stance, and Late Stance. The *x*-axis shows the peak harness-assistance forces, measured based on the amount of forces applied to the harness in the participant's body weight percentage, between slip onset phases (early, mid, and late stance), and groups (younger and older). Error bars represent standard deviation. Dark circles ○ represents the recovery trials and red crosses × represent the fall trials. **(D)** Overlapped histogram demonstrating the number of trials for each group within blocks of 5% of harness assistance. Each block demonstrates counts for both younger and older participants color coded. The red dashed line represents the fall threshold.

Of the 60 slip trials that were analyzed, 40 were classified as recoveries while the remaining 20 were classified as falls. Late stance slips were the most severe for the younger group (six falls) followed by those in early stance (three falls), while early stance slips were most severe for the older group (five falls) followed by late stance (four falls). Mid-stance slips were the least severe in both groups (one fall each) (Figure 2).

According to the optimal data analysis paradigm, all LDA models except frontal plane angular momentum showed moderate effects based on ESS (Table 3). The frontal plane angular momentum LDA model showed a weak effect (Table 3).

**Table 3:** Linear discriminant analysis' (LDA) effect size sensitivity (ESS).

**LDA models**		**Classification** **accuracy**	**Effect size**	
**LDA models**	**Side**		**Effect size** **for sensitivity**	**Interpretation**
Frontal Ftv_CoM_	Standard	73.3%	46.6	Moderate effect
Sagittal Ftv_CoM_	Standard	68.3%	36.6	Moderate effect
Frontal L	Standard	61.7%	23.4	Weak effect
Sagittal L	Standard	65.0%	30.0	Moderate effect

*0.1 < ESS < 24.9 is a weak effect, 25 < ESS < 49.9 is a moderate effect, 50 < ESS < 74.9 is a strong effect, and 75 < ESS < 100 is a very strong effect*.

Q–Q plots showed normal distribution for all variables. However, Shapiro-Wilk tests indicated non-normal distributions in frontal plane peak dominant and minimum non-dominant foot velocity relative to the CoM, as well as peak dominant arm flexion, extension, adduction, and abduction angular momentum and peak non-dominant arm flexion, abduction, and adduction angular momentum.

Backward stepwise elimination completed no more than two steps before finalizing the entered variables for each LDA model (Supplementary Data Sheet 1). Peak frontal plane dominant and peak non-dominant feet velocities relative to the CoM were entered in the frontal plane lower body LDA model as the only significant predictors post-stepwise analysis (*p* < 0.001; Supplementary Data Sheet 1). In the sagittal plane, only peak dominant (*p* = 0.030) and minimum non-dominant (*p* = 0.011) feet velocities relative to the CoM were entered in the sagittal plane lower body LDA model due to statistical significance post-stepwise elimination (Supplementary Data Sheet 1). For the upper body LDA models, peak non-dominant arm adduction angular momentum was entered in the frontal plane model (*p* = 0.006; Supplementary Data Sheet 1), while peak trunk backward angular momentum was entered in the sagittal plane model (*p* = 0.022; Supplementary Data Sheet 1).

**Figure 3:**
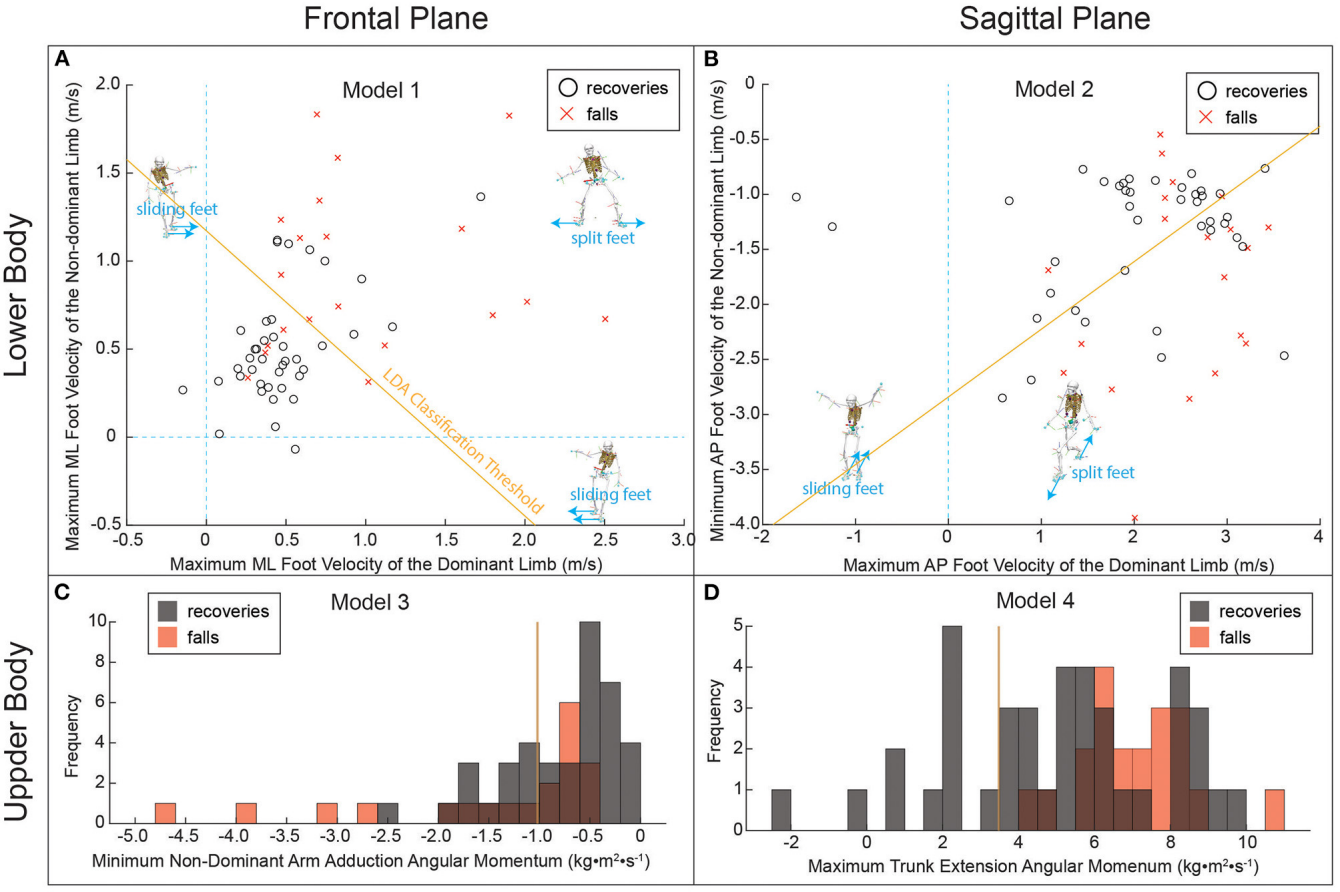
Classification models to discriminate slip and fall trials from recoveries. Four LDA classification models were generated through a process of stepwise variable selection representing kinematic measurements during the slip in either the frontal plane or sagittal plane, and either the lower body or upper body. Solid yellow lines represent the LDA classification threshold where the LDA score is equal to zero. Dashed blue lines represent the separation between slip types (sliding feet or split feet). **(A)** Model 1 demonstrated the highest classification accuracy (73.3%). Falls occurred more often when slipping feet had greater maximum mediolateral velocity. **(B)** Model 2, derived solely from lower body sagittal plane kinematic measurements, demonstrated the second highest classification accuracy (accuracy = 68.3%). **(C)** Model 3 did not accurately discriminate falls from recoveries (accuracy = 61.7%) beyond the null accuracy (66.7%). **(D)** Model 4 demonstrated high sensitivity by correctly classifying all fall trials, but the overall accuracy was low (65.0%) due to the large number of recovery trials with maximum trunk extension velocity above the LDA classification threshold.

Lower body frontal plane predictors were better classifiers of falls and recoveries compared to sagittal plane predictors, supporting our first hypothesis (Figure 3, Supplementary Presentation). Specifically, peak frontal plane dominant and non-dominant feet velocities relative to the CoM (LDA Classification Accuracy = 73.3%, Wilks' Lambda = 0.696, *p* < 0.001) was able to classify both falls and recoveries better than peak sagittal plane dominant and minimum sagittal plane non-dominant feet velocities relative to the CoM (LDA Classification Accuracy = 68.3%, Wilks' Lambda = 0.853, *p* = 0.011). However, sagittal plane upper body predictors were better classifiers of falls and recoveries compared to frontal plane predictors, which does not support our first hypothesis (Figure 3, Supplementary Presentation). The sagittal plane upper body model was able to better classify falls and recoveries using trunk backward angular momentum (LDA Classification Accuracy = 65%, Wilks' Lambda = 0.913, *p* = 0.022) than the frontal plane model using peak non-dominant arm adduction angular momentum (LDA Classification Accuracy = 61.7%, Wilks' Lambda = 0.875, *p* = 0.006). Our second hypothesis was fully supported since peak frontal plane dominant and non-dominant feet velocities relative to the CoM classified both falls and recoveries with higher significance and accuracy (LDA Classification Accuracy = 73.3%, Wilks' Lambda = 0.696, *p* < 0.001) compared to peak non-dominant arm adduction angular momentum (LDA Classification Accuracy = 61.7%, Wilks' Lambda = 0.875, *p* = 0.006). The three variables that were mentioned to answer our second hypothesis were the only significant predictor variables that were included in the LDA classifier post stepwise elimination for each model: (1) sagittal & frontal feet velocities relative to the CoM, and (2) sagittal & frontal upper body angular momentum.

## Discussion

The objective of this study was to examine the importance of frontal plane biomechanics in both testing (e.g. in-lab slips) and analyses (e.g. biomechanical models) when individuals are exposed to unconstrained bilateral slips. Overall, models using peak frontal plane feet velocities relative to the CoM classified falls and recoveries more accurately than models using the same measurement in the sagittal plane and more accurately than models informed by upper body angular momentum in either vertical plane. However, peak sagittal plane trunk backward angular momentum was able to classify both slip-related falls with perfect sensitivity but low specificity, resulting in low overall accuracy.

Before we can discuss and explain our findings, we first need to highlight the impact of low friction slippery surfaces on gait. Walking on a slippery surface changes our gait kinetics, kinematics, and muscle activation patterns (29). Moreover, it was previously demonstrated that walking on a slippery surface affects both biomechanics through changes in reactive gait during the initial step on a slippery surface (38–41), as well as motor control through changes in proactive behavior after extended exposure to the slippery surface (29, 32). Here, we focused our analysis on the initial slip exposure for each slip onset phase, however we acknowledge that there are likely learning effects after the initial slip regardless of the difference in onset phase. Specifically, Cappellini et al. (29) demonstrated that alongside an increase in frontal plane arm excursions, with an observable higher shoulder adduction that resulted in higher frontal plane trunk angles, the most noticeable difference to gait when walking on a slippery surface was a reduction in shear forces while sustaining normal forces. Interestingly, they show that the mean AP shear forces were reduced near zero during stance phase, while ML shear forces were applied laterally throughout stance phase. This indicates a strong preference to maintain a non-zero step width and a mediolateral oscillatory pattern in the CoM driven by ML shear forces, which becomes extremely challenging with low friction while still maintaining forward momentum. An alternative explanation could be that slight deviations in frontal plane slips could be better controlled than deviations in sagittal plane slips. Such findings could explain the superior accuracy of the frontal plane lower body model based on dominant and non-dominant feet velocities relative to the CoM in classifying both falls and recoveries.

We hypothesized that frontal plane feet velocities relative to the CoM would be a better predictor of falls and recoveries than sagittal plane feet velocities relative to the CoM and sagittal and frontal plane upper body angular momentum, due to the fact that upper body angular momentum is regulated by base of support placement relative to the CoM accompanied by normal and shear ground reaction forces (24) across the gait cycle. Here we show that it is important to maintain dominant and non-dominant foot velocities similar to that of the CoM and to each other in both the sagittal and frontal planes. In fact, frontal plane foot motion relative to the CoM and to one another appears to be of most significance in achieving recoveries and a potential indicator of fall severity. It is difficult to relate our results to previous literature since we are the first to induce simultaneous bilateral slips. However, Troy et al. (21) showed that if both the sagittal plane velocity and the lateral distance of the slipping foot relative to the CoM were to drop to zero, the probability of recovery would increase by 16.6 and 26.5%, respectively.

In our model, we included the peak velocity of both feet relative to the CoM and to each other which gives the model more flexibility and avoids multicollinearity, potentially providing a better capability to discriminate between falls and recoveries. Frontal plane lower body models revealed two biomechanical behaviors that were associated with falls: (1) frontal plane sliding feet, where both feet travel in the same direction (ipsilaterally/contralaterally) and opposite to the CoM, and (2) frontal plane split feet, where both feet travel faster than the CoM and opposite to each other. Sagittal plane lower body models, on the other hand, revealed two biomechanical behaviors that are similar to those described in past literature (17, 19, 21): (1) sagittal plane split feet, where the leading foot travels faster than the CoM and opposite to the trailing foot, which travels opposite to both, and (2) sagittal plane forward sliding mechanism, where both feet travel with a velocity similar to that of the CoM. However, we believe that foot motion relative to the CoM is not the only contributor to falls and recoveries, and that upper body biomechanics play a crucial role in redistributing sagittal and frontal plane angular momentum from the trunk to the arms.

Hence, we presented our second hypothesis and demonstrated that the peak sagittal and frontal plane angular momentum of the trunk and non-dominant arm during the slipping timeline, respectively, were significant in classifying falls and recoveries. Despite extensive literature on the significance of peak trunk extension angle to both falls and recoveries (9, 13, 17, 19, 23, 29, 42–45), our variable selection process generated a model based on arm adduction angular momentum. This would indicate that bracing for impact by increasing arm adduction angular momentum is associated with higher fall probability. Such an outcome could be due to the failure to reduce frontal plane whole-body angular momentum during a lateral fall, corresponding to an increase in trunk angular momentum that could be reduced by rapidly abducting the arm on the falling side. Without applying torques through ground reaction forces, increasing the moment of inertia by raising the arms while conserving whole-body angular momentum will decrease angular velocity, providing more time to alter the base of support. Even if angular momentum is not constant (i.e. torques applied through ground reaction forces), increasing the moment arm will also increase the moment of inertia, in turn increasing the resistance to the body's rotation.

An alternative explanation may be that fall directions were such that the non-dominant limb was more often in the best position to assist through abduction but failed to do so. Motor timing coordination theory (46), which characterizes individuals' abilities to recognize and produce motor actions with an appropriate spatial and temporal manner, could explain why we found a significant effect of non-dominant arm adduction angular momentum and not of that of the dominant arm. Failure to deliver a coordinated performance could be due to either the task being performed, or the limb used to execute the task. Usage of the non-dominant limb has been shown to offer less advantages in terms of movement rate (47), temporal consistency (48), and strength (49). Moreover, it was previously shown that increased usage of and strength in the dominant limb showed superior central nervous system activation in the motor cortex (50), and higher motor unit recruitment and firing rates within the spinal circuitry (51). Thus, maybe rehabilitation protocols should focus on improving task specific motor control of individuals' non-dominant limbs.

The small sample size used in this study is a potential limitation, as the results in the current paper are an interim analysis of data from a larger ongoing study designed to test learning effects. The power analyses were completed for the whole project which is a limitation of this study. But the effect sizes presented in Table 3 show moderate power for all models except for the frontal plane angular momentum model. Three trials labeled as a recovery may have involved some limited harness assistance <30%. Limiting the classification of the trial outcome to either fall or recovery is a limitation since an intermediate harness assistance outcome was not considered in this analysis. Excluding these three trials has a small effect on the model accuracies (New LDA: Frontal Lower: 77.2%, Sagittal lower: 64.9%, Frontal upper: 63.2%, Sagittal lower: 63.2%), but does not affect the findings, hence was the rationale behind not excluding them from the analyses. Upper body angular momentum did not involve the neck and head, which could be a limitation as they can contribute a small but significant amount to the angular momentum. The non-normality observed in this study is a limitation that could be due to the number of kinematic factors added in the analyses (20 factors). Future studies should use statistical methods that do not rely on normality, and if discriminant analysis will be used, future studies should consider using penalized (52) and quadratic (53) discriminant analyses. Despite the investigator having extensive training and experience in triggering the WASP at the specific stance phases to account for the human reaction time delay, triggering the WASP through online visual observation is vulnerable to human error. Fall history was not used as an exclusion criterion in order to include a diverse set of adults from the community. We tested the dynamic coefficient of friction between the interface of the WASP's insole and both the outsole and lab tiles by assuming that friction coefficients provided by the WASP followed an idealistic linear model of friction, however it is possible that this assumption may be violated under larger loads such as those during walking. However, friction is likely to stay linear as we previously tested static coefficient of friction of the WASP under a range of simulated body weights and it did not change (7). Learning effects may arise after exposure to the first slip despite being exposed to different types of slip onset phases during stance, hence this could be a limiting factor, especially since literature reported biomechanical and motor adaptation after a single exposure to sagittal induced (54, 55) and unconstrained induced (56) in-lab slips. The previously mentioned studies did not report the exact resting period between each slip trial, and despite the resting period (5-min) was controlled in this study, it is uncertain if such resting period is sufficient to exclude proactive adaptation. Another limitation of using data driven models to determine kinematic factors associated with falls in a stepwise classification analysis is that, when the resulting models are low accuracy, the factors chosen during the variable selection process may be arbitrary and other factors are likely to be chosen if the same analysis was repeated with a different sample of participants (57). Furthermore, using models cannot determine whether those factors are causally associated with falls. Further study of the factors we identified here is warranted to determine if they are modifiable and causally related to falling from unconstrained slips. Future studies may gain additional insight into causal relationships between potential biomechanical factors and fall outcomes by examining muscle activation during the slipping timeline. Future studies should investigate the coordination patterns between the upper and lower body in both frontal and sagittal planes. Younger and older adults were combined in the same LDA analyses for three main reasons; (1) to investigate the significance of frontal plane biomechanics on in-lab unconstrained slip induced falls regardless of the group, (2) both younger and older adults showed similar falls and recoveries, (3) this study was not focused on developing task specific training to avoid falls during unconstrained bilateral slips. Future studies should run a separate LDA analyses for older and younger adults to determine the appropriate task specific training to avoid falls and achieve recoveries when exposed to unconstrained bilateral slips.

## Conclusion

Overall, our findings demonstrate that unconstrained simultaneous bilateral slips disrupt dynamic stability and generate falls during slips that are initiated at early, mid, and late stance phase of the dominant foot. Specifically, late stance slips administered to the dominant foot (with the non-dominant foot experiencing an early stance slip simultaneously) led to the highest fall incidence in young adults, while the reverse led to the highest fall incidence in older adults. Comparisons between classification models indicated that frontal plane kinematics enabled the highest accuracy. The clinical implication derived from these results describes the behavior of younger and older adults during unconstrained simultaneous bilateral slips and stresses the importance of incorporating the frontal plane aspect during testing and analyses when individuals are exposed to unconstrained slips.

## Data Availability Statement

The raw data supporting the conclusions of this article will be made available by the authors, without undue reservation.

## Ethics Statement

The studies involving human participants were reviewed and approved by University of Nebraska Medical Center Institutional Review Board (IRB). The patients/participants provided their written informed consent to participate in this study. Written informed consent was obtained from the individual(s) for the publication of any potentially identifiable images or data included in this article.

## Author Contributions

AO involved in design, data acquisition, data analysis, data visualization, statistical analyses, data interpretation, preparing, writing, editing, and reviewing the original manuscript draft. CR involved in data acquisition, data interpretation, and editing and reviewing the manuscript draft. NH secured funding, involved in design, data analysis, editing and reviewing the manuscript draft, and supervising the study. All authors read and approved the final manuscript version.

## Funding

This work was supported by the National Institutes of Health; NIH 2P20 GM109090-06 and NIH R15AG063106-01.

## Conflict of Interest

The authors declare that the research was conducted in the absence of any commercial or financial relationships that could be construed as a potential conflict of interest.

## Publisher's Note

All claims expressed in this article are solely those of the authors and do not necessarily represent those of their affiliated organizations, or those of the publisher, the editors and the reviewers. Any product that may be evaluated in this article, or claim that may be made by its manufacturer, is not guaranteed or endorsed by the publisher.
