# Supplementary material for: Severity of Unconstrained Simultaneous Bilateral Slips: The Impact of Frontal Plane Feet Velocities Relative to the Center of Mass to Classify Slip-Related Falls and Recoveries
Source: Front Public Health. 2022 Jul 11;10:898161. doi: 10.3389/fpubh.2022.898161 (PMC9309647; doi:10.3389/fpubh.2022.898161)
Supplement: Supplementary file 2 [file Image_1.pdf]

FullBody_20191113

Marker Placement

Body Segment	Name	Description
Head	1. RHD1<br>2. MHD2<br>3. LHD3<br>4. AHD4	1/3. Above the left/right ears, superficial to the left/right temple axis.<br>2. Mid-distance between nasal to inion. At the tip of the head. Highest height.<br>4. tracking marker; Placed at the forehead anterior to the MHD2 along the same axis
Upper Torso	5. STRN<br>6. XYPH<br>7. C007<br>8. T008<br>9. LACR<br>10. RACR	5. Superficial to the sternal notch.<br>6. Superficial to the xiphoid process.<br>7. Superficial to palpable prominence of C7.<br>8. Superficial to the spinous process below a midpoint between the scapular tips.<br>9/10. Superficial to palpable prominence of the acromion processes.
Upper Arm	11. LSHA<br>12. LSHP<br>13. LUP1<br>14. LUP2<br>15. LUP3<br>16. LUP4<br>17. LEBL<br>18. LEBM<br>19. RSHA<br>20. RSHP<br>21. RUP1<br>22. RUP2<br>23. RUP3<br>24. RUP4<br>25. REBL<br>26. REBM	11/19. Anterior to the glenohumeral joint.<br>12/20. Posterior to the glenohumeral joint.<br>13-16/21-24. Clusters: Lateral on the upper arm, approximately midway between the shoulder and elbow.<br>17/25. Lateral aspect of the humerus along the elbow joint line.<br>18/26. Medial aspect of the humerus along the elbow joint line.
Lower Arm	27. LFR1<br>28. LFR2<br>29. LFR3<br>30. LFR4<br>31. RFR1<br>32. RFR2<br>33. RFR3<br>34. RFR4<br>35. LWT1<br>36. LWTR<br>37. LWTU<br>38. RWT1<br>39. RWTR<br>40. RWTU	27-30/31-34.Posterior on forearm proximal to wrist.<br>35-38. Between the distal end of the 2nd and 3rd metacarpals.<br>36/39. Medial aspect of the Radial styloid process.<br>37/40. Lateral aspect of the Ulnar styloid process.
Lower Torso	41. LASI<br>42. RASI<br>43. LPSI<br>44. RPSI	41/42. Superficial on skin surface such that marker body is anterior to prominent anterior edge of iliac crest (anterior to IAS from [3]).<br>43/44. Superficial on skin surface at the most lateral point on the superior tubercle of the iliac crest.

	45. SACR	45. Distal to the mid-point between the PSI markers.
Thigh	46. LGTO<br>47. RGTO<br>48. LTH1<br>49. LTH2<br>50. LTH3<br>51. LTH4<br>52. RTH1<br>53. RTH2<br>54. RTH3<br>55. RTH4<br>56. LKNL<br>57. RKNL<br>58. LKNM<br>59. RKNM	46/47. Superficial to palpable prominence of the greater trochanter.<br>48-51/52-55.Laterally on the thigh approximately mid distance between the knee joint line and the greater trochanter.<br>56/57. Laterally on the knee joint line mid distance from the anterior to posterior knee excluding the patella when viewed from the side.<br>58/59. Medially on the knee joint line mid distance from the anterior to posterior knee excluding the patella.
Shank	60. LSH1<br>61. LSH2<br>62. LSH3<br>63. LSH4<br>64. RSH1<br>65. RSH2<br>66. RSH3<br>67. RSH4<br>68. LANL<br>69. RANL<br>70. LANM<br>71. RANM	60-63/64-67. Laterally on the shank, approximately mid distance between the knee and ankle joints<br>68/69. At most lateral point on lateral malleolus.<br>70/71. At most medial point on medial malleolus.
Foot	72. LCA1<br>73. LCA2<br>74. RCA1<br>75. RCA2<br>76. LHEE<br>77. RHEE<br>78. LMT1<br>79. LMT5<br>80. LTOE<br>81. RMT1<br>82. RMT5<br>83. RTOE	72/77. Medial on the metatarsals approx. mid distance between ANL and MT5.<br>73/75. Lateral on the metatarsal approx. mid distance between the ANM and TOE.<br>76/77. On heel counter at the same height as TOE, centrally when viewing from a posterior position along the long axis of the shoe.<br>78/81. Medially on shoe at a point approximating position of 1st metatarsal head.<br>79/82. Laterally on shoe at a point approximating position of 5th metatarsal head.<br>80/83. At a point approximating position of second metatarsal head on dorsum of shoe.

Total Number of Markers = 83

Total Number of Markers (excluding Clusters) = 49

Total Dynamic trial markers= 75

Blue – Tracking only
Green – Joint definition
Orange – Joint definition, static only
Clear – Not current used

COBRE Slip Study

ID#

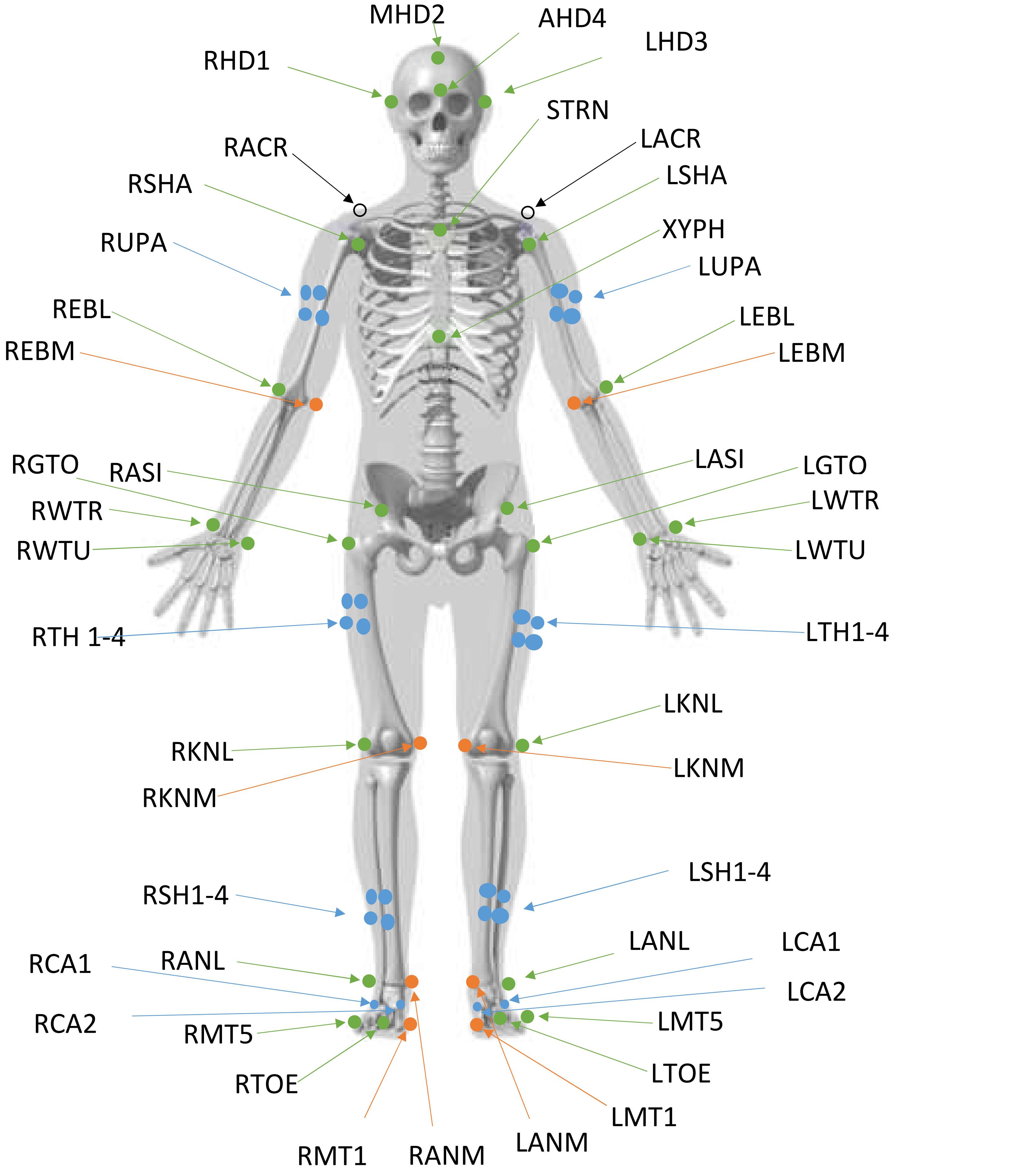

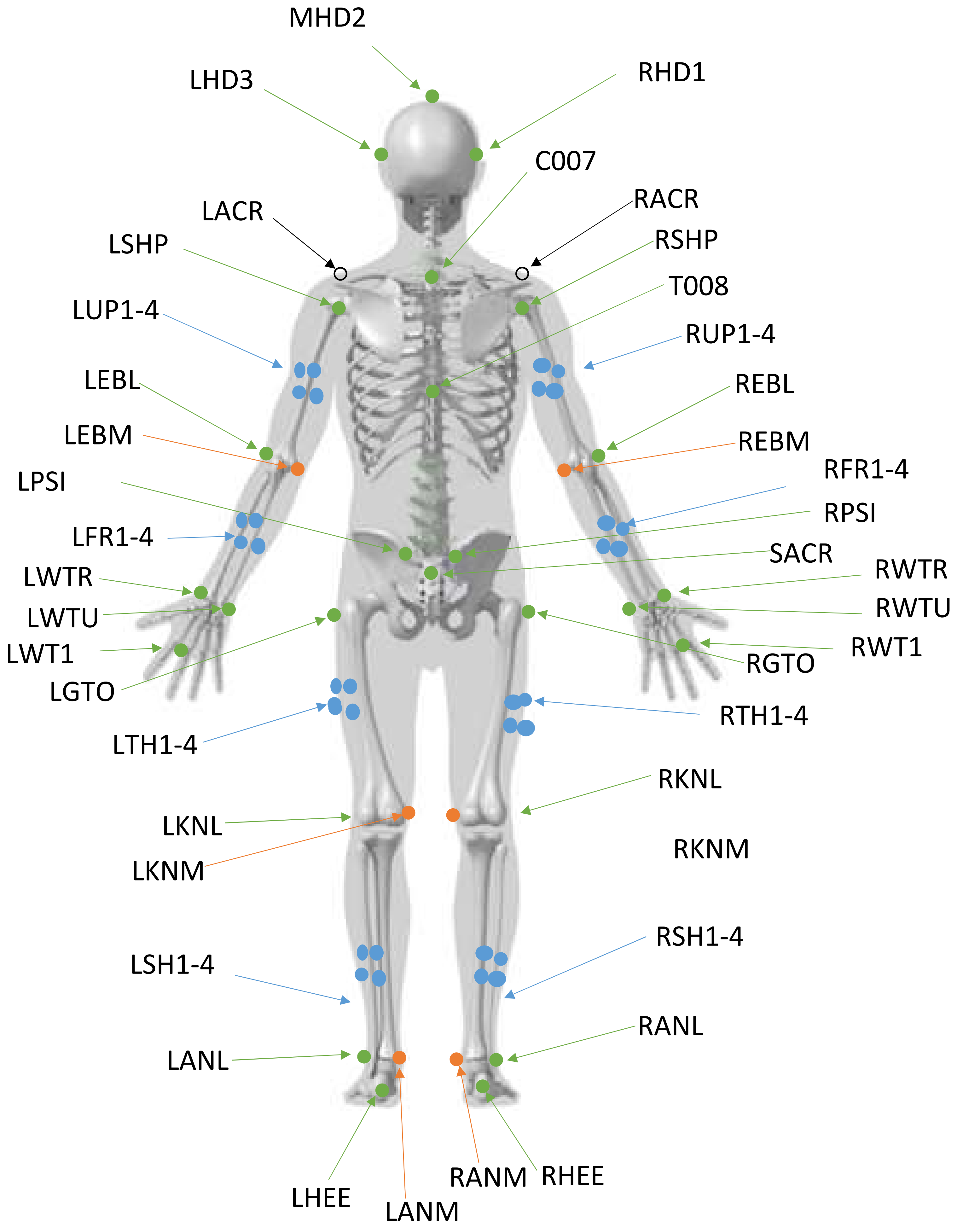
MHD2
LHD3
RHD1
C007
LACR
RACR
LSHP
RSHP
T008
LUP1-4
RUP1-4
LEBL
REBL
LEBM
REBM
RFR1-4
LPSI
RPSI
LFR1-4
SACR
LWTR
RWTR
LWTU
RWTU
LWT1
RWT1
RGTO
LGTO
RTH1-4
LTH1-4
RKNL
LKNL
RKNM
LKNM
RSH1-4
LSH1-4
RANL
LANL
RANM
RHEE
LHEE
LANM
