# Supplementary material for: Severity of Unconstrained Simultaneous Bilateral Slips: The Impact of Frontal Plane Feet Velocities Relative to the Center of Mass to Classify Slip-Related Falls and Recoveries
Source: Front Public Health. 2022 Jul 11;10:898161. doi: 10.3389/fpubh.2022.898161 (PMC9309647; doi:10.3389/fpubh.2022.898161)
Supplement: Supplementary file 3 [file Presentation_1.PPTX]

## Slide 1
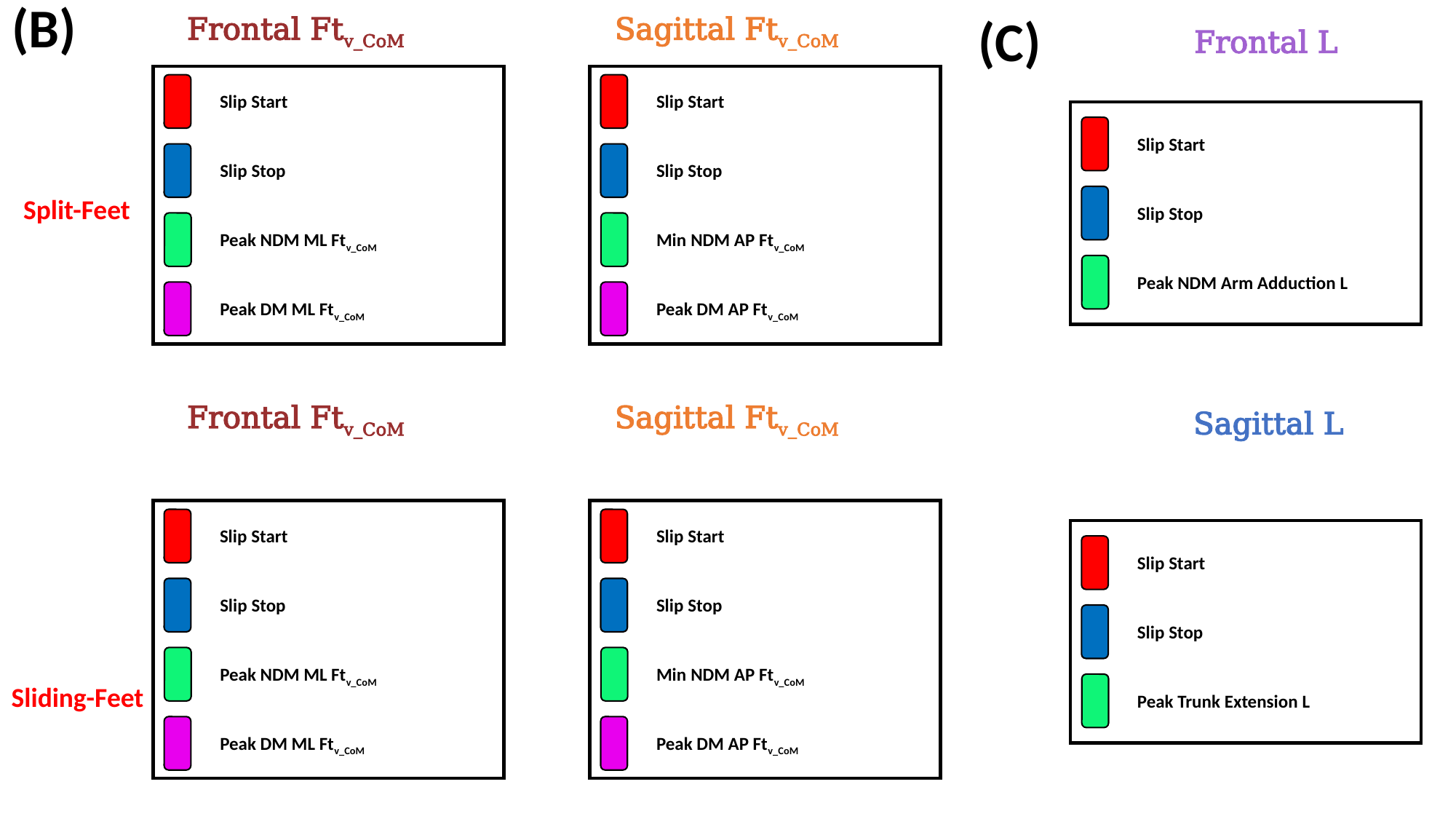

(B)
(C)
Frontal Ftv_CoM
Sagittal Ftv_CoM
Frontal L
Slip Start
Slip Start
Slip Start
Slip Stop
Slip Stop
Split-Feet
Slip Stop
Peak NDM ML Ftv_CoM
Min NDM AP Ftv_CoM
Peak NDM Arm Adduction L
Peak DM ML Ftv_CoM
Peak DM AP Ftv_CoM
Frontal Ftv_CoM
Sagittal Ftv_CoM
Sagittal L
Slip Start
Slip Start
Slip Start
Slip Stop
Slip Stop
Slip Stop
Peak NDM ML Ftv_CoM
Min NDM AP Ftv_CoM
Sliding-Feet
Peak Trunk Extension L
Peak DM ML Ftv_CoM
Peak DM AP Ftv_CoM

## Slide 2
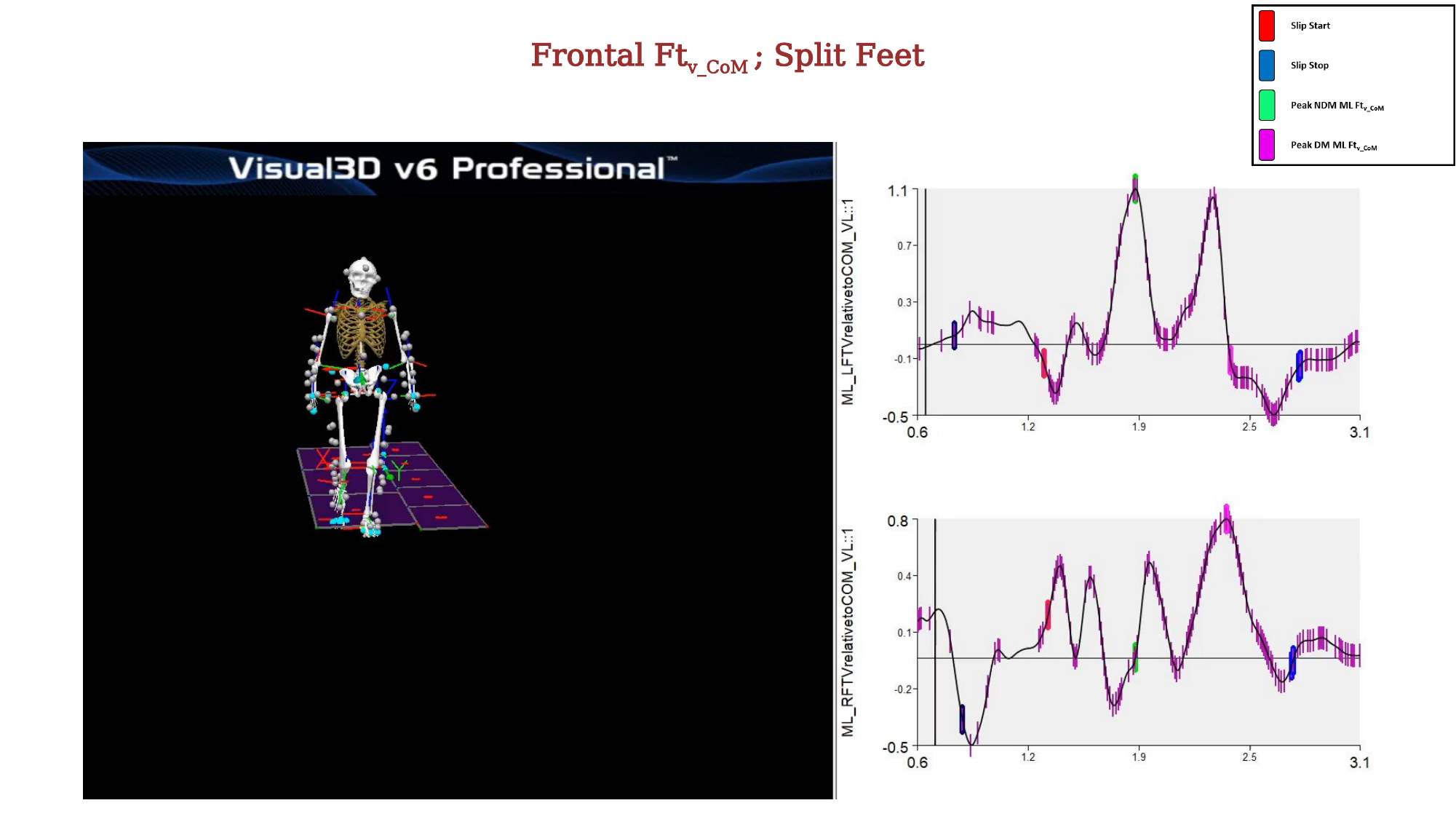

Frontal Ftv_CoM ; Split Feet

## Slide 3
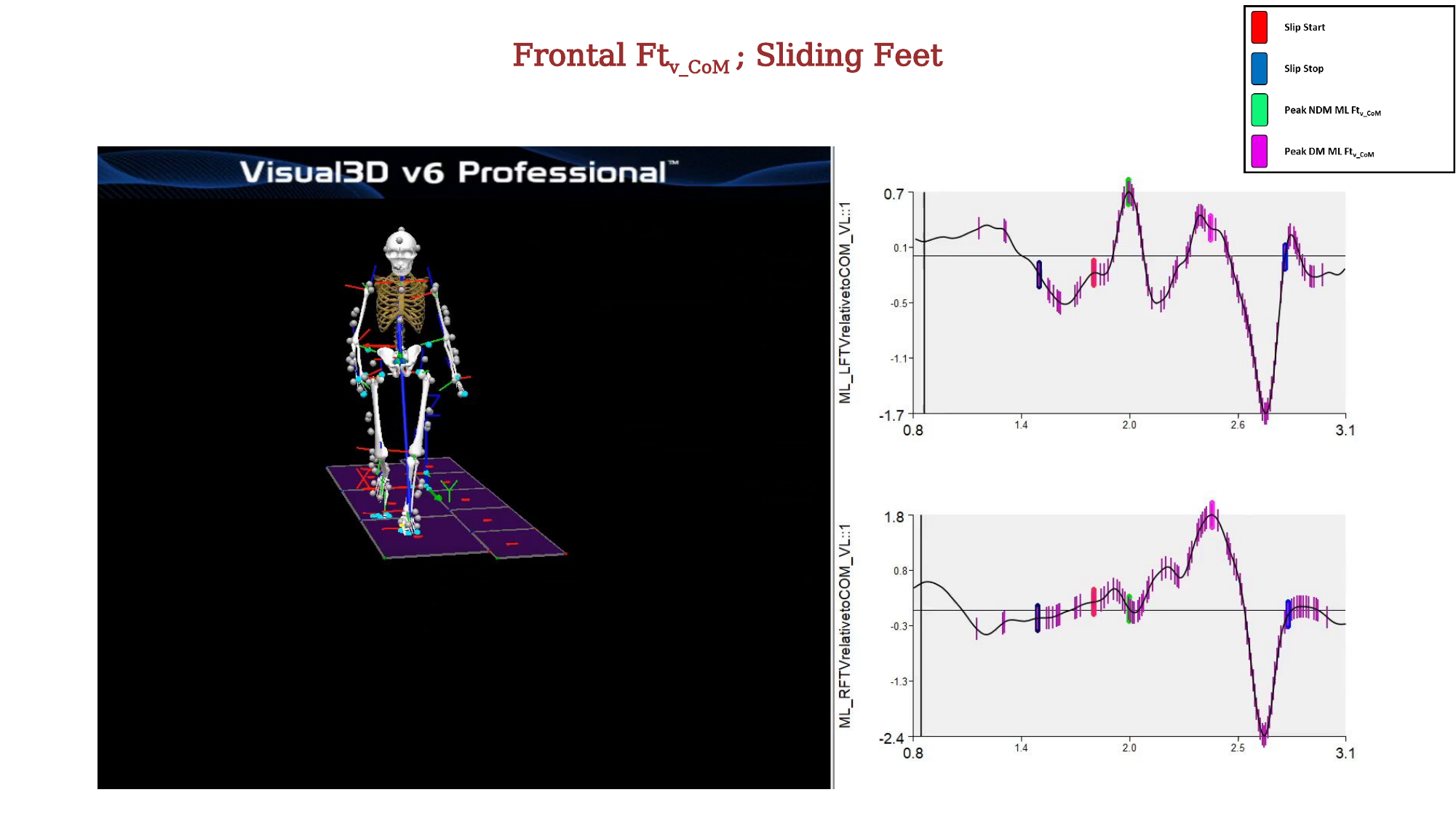

Frontal Ftv_CoM ; Sliding Feet

## Slide 4
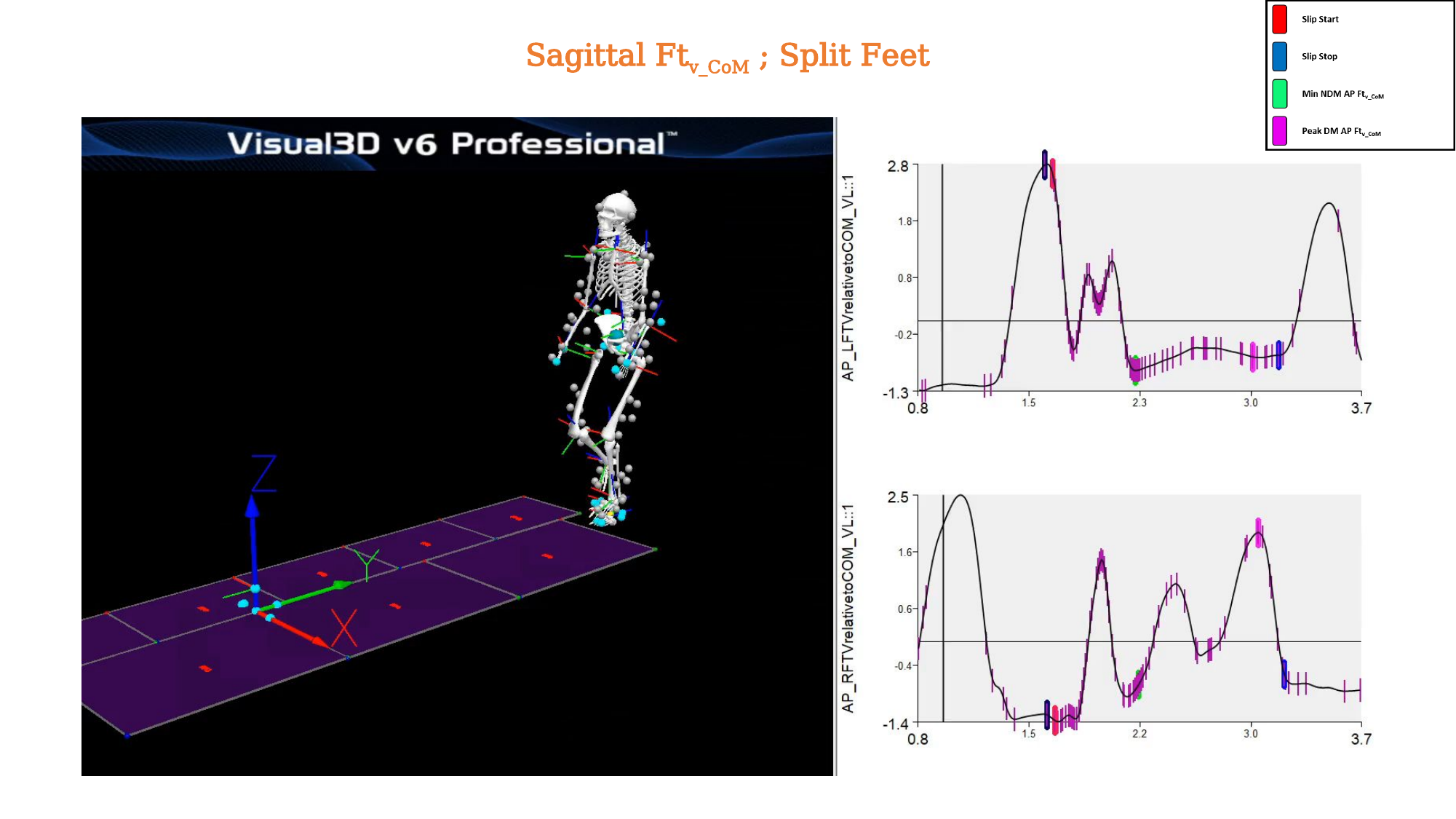

Sagittal Ftv_CoM ; Split Feet

## Slide 5
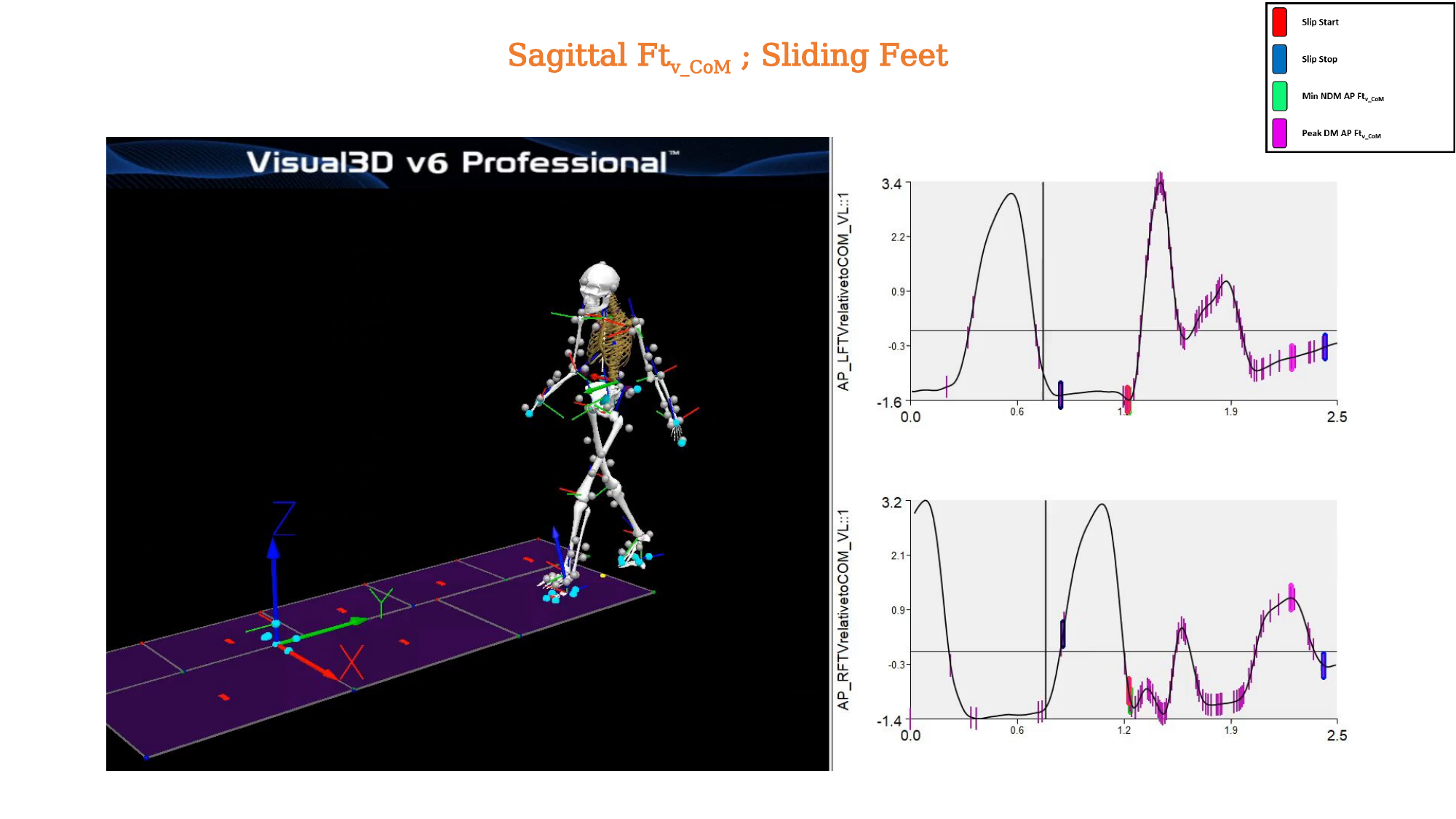

Sagittal Ftv_CoM ; Sliding Feet

## Slide 6
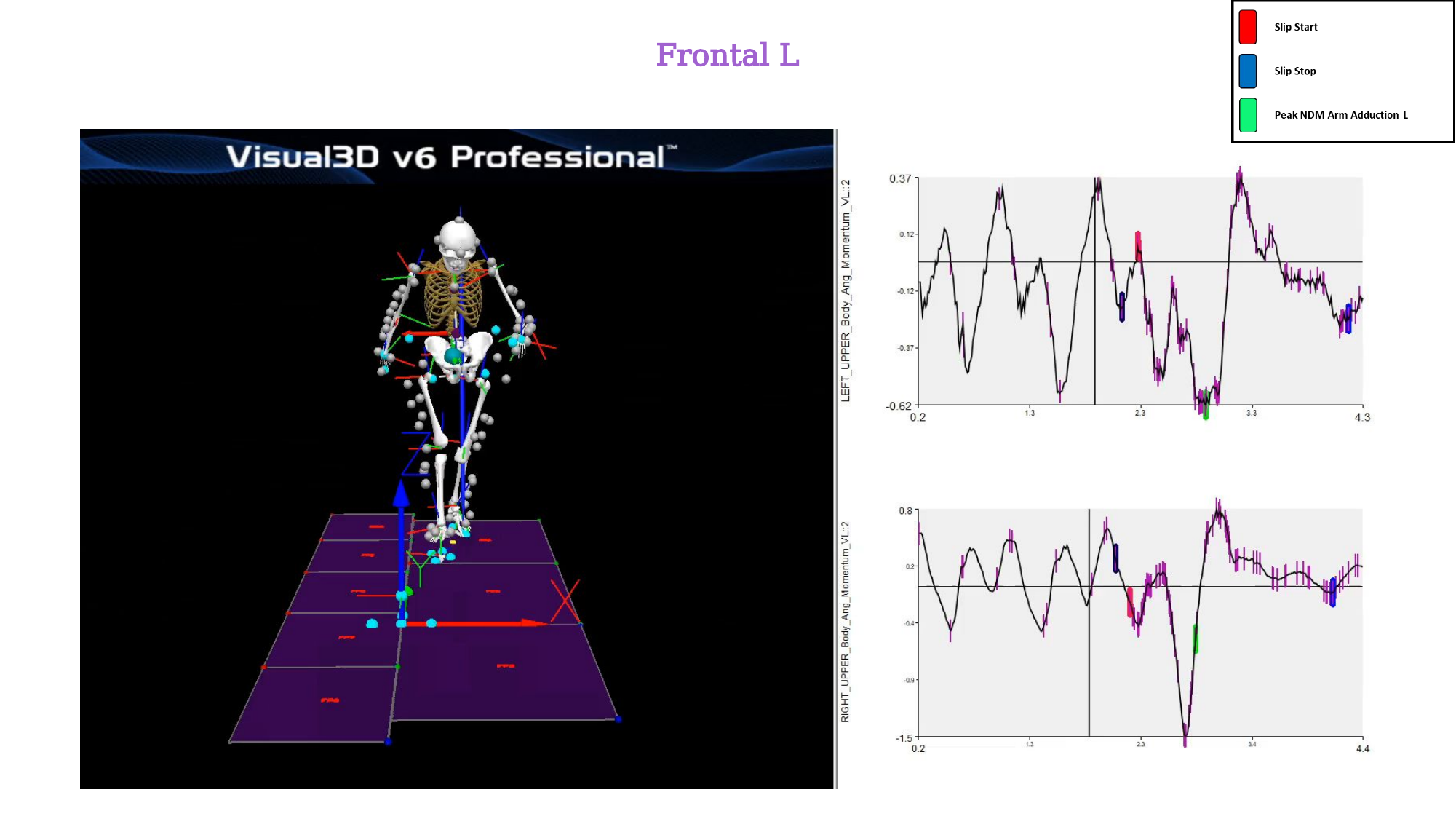

Frontal L

## Slide 7
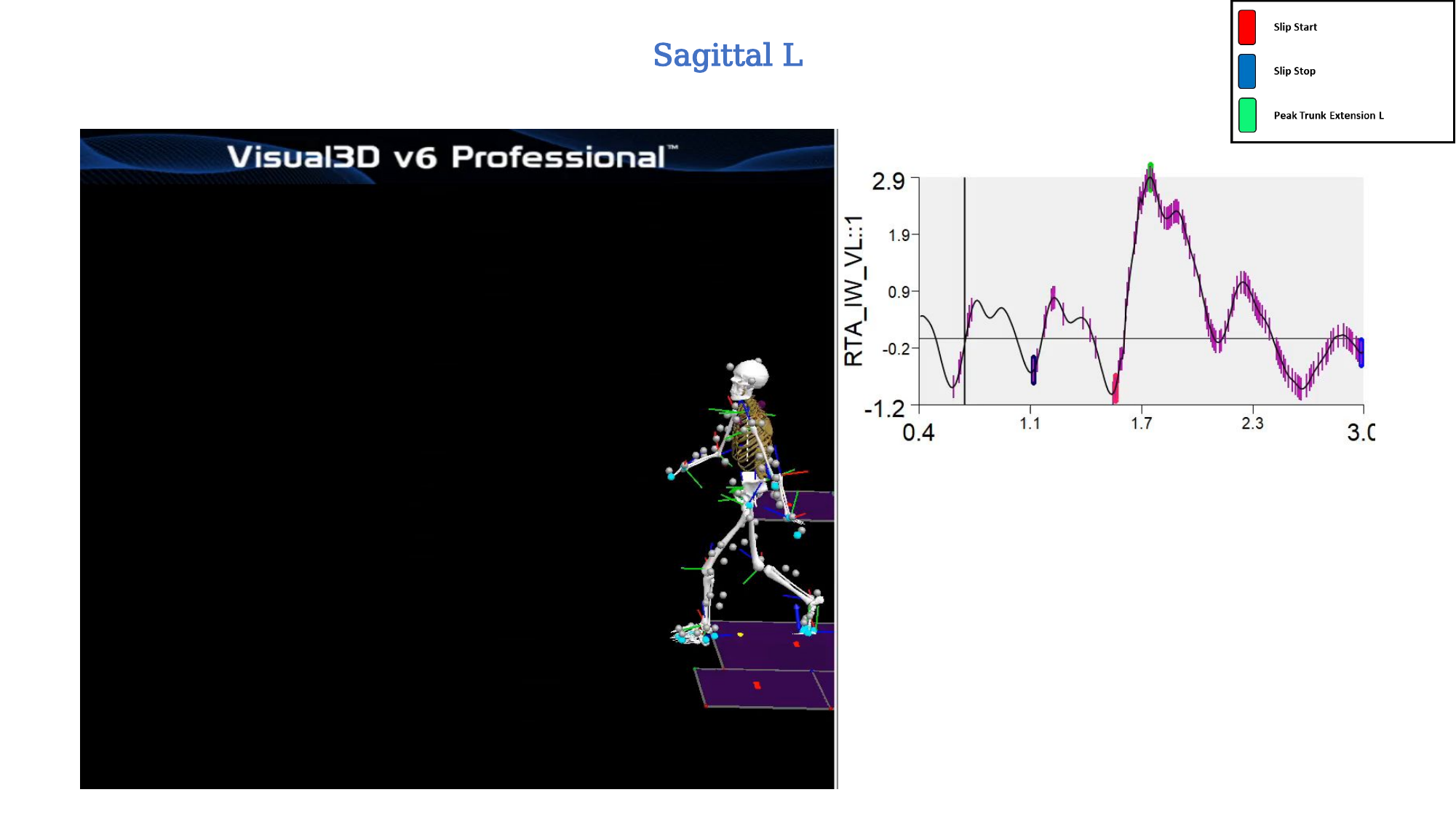

Sagittal L
